# The Moderating Effect of Nursing Practice Environment on the Relationship between Clinical Nurses’ Sleep Quality and Wellness

**DOI:** 10.3390/ijerph17197068

**Published:** 2020-09-27

**Authors:** Kyung Jin Hong, Youngjin Lee

**Affiliations:** 1 Department of Nursing, Semyung University, 65 Semyung-ro, Jecheon-si, Chungcheongbuk-do 27136, Korea; unit1@snu.ac.kr; 2 College of Nursing, Ajou University, 164 World cup-ro, Yeongtong-gu, Suwon 16499, Korea

**Keywords:** sleep quality, nursing practice environment, wellness, moderating effect, nursing productivity

## Abstract

This study examined the moderating effect of nursing practice environment on the relationship between clinical nurses’ sleep quality and wellness. The wellness of clinical nurses is a direct outcome of individual-level health behaviors and organizational environmental factors. This study was a cross-sectional analysis. Participants were clinical nurses recruited using convenience sampling. The Nurse Practice Environment Scale, Wellness Index, and Pittsburgh Sleep Quality Index, Korean version (PSQI-K) were used. Data collected from 1874 nurses were analyzed using descriptive statistics and hierarchical multiple regression analyses. A total of 95.3% of the participants were women, and the mean age was 28.8 years. Further, 42.4% of the participants had a nursing career of 5 years or longer. The mean score for nursing practice environment was 2.24 and the mean PSQI-K score was 9.39. Nurses with less than 1 year of experience reported lower wellness scores. The wellness scores decreased with poorer sleep quality, and a more positive evaluation of the nursing practice environment predicted higher levels of wellness. Nursing practice environment had a moderating effect on the negative association of nurses’ poor sleep quality with their wellness. Regarding management, individual strategies for nurses’ well-being and organizational improvement policies may improve the nursing work environment.

## 1. Introduction

Most nurses work in shifts due to the necessity to provide 24-h direct care for patients. However, this type of work schedule may have a negative impact on nurses’ physical and emotional wellness [1]. Prior studies have shown that shiftwork reduces nurses’ sleep quality and is associated with an increase in medical errors due to attention deficit and delays in reaction time [2]. Thus, managing the sleep quality of nurses is extremely important to providing high-quality nursing services and ensuring the safety of patients [3,4,5]. Because sleep is an essential aspect of well-being, lack of sleep can contribute to tiredness, anxiety, aggression, nervousness, and tension [6,7]. Various factors affect sleep quality, including personal characteristics and practice environmental factors, such as job demands and job control [8], ability to control adverse events, and social support [9]. The nursing practice environment can affect nurses’ emotional stress and job outcomes [10]. Nursing practice environment is defined as the organizational characteristics of a work setting that facilitate or constrain professional nursing practice [11]. In a past study, nurse managers’ ability, leadership, and support components of the nursing practice environment were predictors of nurses’ fatigue, and fatigue was associated with sleep quality [12]. However, studies that shed light on the relationship between sleep quality and nursing practice environment are lacking, and further investigation is needed. In this study, we aimed to consider personal characteristics related to the sleep quality of nurses, in addition to examining the characteristics of nursing medical institutions, such as nursing practice environment.

Nursing practice environment is a concept that includes the physical environment, interactions between individuals, and policy aspects [13]. This can be characterized by participation in hospital affairs; nursing foundations that ensure quality of care; nurse manager abilities, leadership, and support of nurses; staffing and resource adequacy; and collegial nurse–physician relationships [11]. These factors can affect job satisfaction, patient safety, and the stability of nursing staff [14]. A good nursing practice environment is indicative of a supportive medical institution and is expected to decrease the negative effects of poor sleep quality. In this environment, it is possible for nurses to receive assistance with errors or physical difficulties related to poor sleep quality.

Wellness is a condition whereby the emotional, social, intellectual, and psychological states stay in harmony with each another [15]. Wellness can not only enhance an individual’s quality of life but also promote productivity by reducing absences or presenteeism, which occurs when extreme stress at the workplace negatively affects the performance of an employee on a job. Moreover, wellness also has a positive effect on the quality of care services provided to patients [16]. Because sleep quality is an important factor that affects the wellness of nurses [17], these two concepts should be considered together.

As such, this study examined differences in sleep quality, nursing practice environment, and wellness according to the general demographic characteristics of nurses, in addition to identifying the wellness factors that benefit nurses. We hypothesized that although poor sleep quality is negatively associated with nurses’ wellness, a positive nursing practice environment with sufficient support can have a moderating effect on the negative consequences of poor sleep quality. To date, only a few studies have examined the impact of nursing practice environment on quality of life, such as nurses’ wellness, and the relationship between them. In particular, confirming that nursing practice environment has a buffering effect in the impact of an important health factor such as sleep quality on wellness would provide valuable evidence to support the need to improve the nursing practice environment. Therefore, this study sought to investigate the moderating effect of nursing practice environment on nurses’ sleep quality and its relationship with wellness.

## 2. Materials and Methods

### 2.1. Study Design

A descriptive, cross-sectional study design was employed using secondary analysis of a parent study, the Compassion Cohort Study (AJIRB-SBR-SUR-17-386). The Compassion Cohort Study is a longitudinal research project evaluating changes in nursing students’ and registered nurses’ compassion competence, including the effects of these changes on nursing profession and quality of care. The present study was designed to explore the relationships among sleep quality, nursing practice environment, and wellness according to the sociodemographic characteristics of nurses, and to identify the moderating effect of nursing practice environment on the relationship between clinical nurses’ sleep quality and wellness.

### 2.2. Setting and Participants

In total, 2041 study participants were recruited using convenience sampling among clinical nurses who were currently working in hospitals and provided written consent. The inclusion criteria for this study were as follows: (a) Nurses who were working at hospitals with a capacity of over 100 beds in Korea; (b) nurses who were providing direct care for patients; and (c) nurses who had more than three months of nursing experience. Nurses were excluded from the study if they were involved in administrative tasks rather than providing direct patient care.

### 2.3. Data Collection and Procedure

This study was conducted after obtaining approval from the Institutional Review Board (No.: AJIRB-SBR-MDB-18-552). Clinical nurses were recruited via poster advertisements, homepage banners for research, social media, and word of mouth. The participants were informed about the study purpose and procedures and were requested to complete the questionnaire. They were provided informed consent forms, which were signed and returned along with the questionnaires. All participants remained anonymous. In total, 2041 nurses agreed to respond to the survey, which was conducted between February and October 2018. Of the 2041 questionnaires, 1937 were completed, resulting in a 94.9% response rate. We excluded 63 questionnaires from the analysis due to missing responses to some questions. Thus, data from 1874 nurses were included in the analyses.

### 2.4. Measures

The Nurse Practice Environment Scale (PES-NWI), which was used to evaluate the nursing practice environment in this study, was developed by Lake [11] and translated into Korean by Cho, Choi, Yoo, and Lee [18]. This scale comprises 29 items across five subscales: Nurses’ participation in hospital affairs (nine items); foundation for the quality of care (nine items); staffing and resource adequacy (four items); nurse manager ability, leadership, and support of nurses (four items); and collegial nurse–physician relationship (three items). Each item is rated on a four-point Likert scale from 1 (“strongly disagree”) to 4 (“strongly agree”), with a higher score indicating a better environment. The overall score for the PES-NWI is the average value of each item, and the Cronbach’s alpha coefficient was 0.94.

Wellness was measured using the Wellness Index (WI), developed and validated in Korean by Choi, Son, Kim, and Ha [19]. This self-report questionnaire comprises 18 items across five wellness dimensions: Physical (four items), emotional (five items), social (three items), cognitive (three items), and occupational (three items). Each item is rated on a five-point Likert scale ranging from 1 (not at all) to 5 (extremely true). The total and subscale scores are calculated by averaging the scores on all items within each dimension. A higher score indicates a higher level of wellness. The Cronbach’s alpha coefficients were 0.91 for the original WI and 0.91 in this study.

Sleep quality and sleep patterns were assessed by the Korean version of the Pittsburgh Sleep Quality Index (PSQI-K), translated and validated by Sohn, Kim, Kee, and Cho [20], which was based on the original PSQI developed by Buysse, Reynolds, Monk, Berman, and Kupfer [21]. This questionnaire comprises 18 self-report items across seven components: Subjective sleep quality, sleep latency, sleep duration, habitual sleep efficiency, sleep disturbances, use of sleep medication, and daytime dysfunction. Each item is rated on a four-point Likert scale ranging from 0 (never) to 3 (more than three times a week). The global score of the PSQI-K ranges from 0 to 21, with a higher score indicating poorer sleep quality. Based on the criteria set by Buysse et al. [21], the Cronbach’s alpha coefficients were 0.84 for the original PSQI-K [20] and 0.70 in this study.

The questions were related to general characteristics, such as gender, education level, marital status, the region of working institution, and tenure as a registered nurse (RN). Moreover, one item questioned perceived health status, which was rated on a scale from 1 (very unhealthy) to 4 (very healthy), based on which participants were categorized into two groups: Healthy (very healthy to healthy) or not healthy (unhealthy to very unhealthy).

### 2.5. Analysis

The data were analyzed using SPSS version 23.0 (IBM Corp., Armonk, NY, USA). The level of significance was set to *p* ≤ 0.05. Descriptive analyses were used to calculate the means ± standard deviation of the major factors, and *t*-tests and one-way analysis of variance (ANOVA) were performed to examine differences in the means among the factors according to the sociodemographic characteristics. Additionally, a post-hoc test was conducted using the Scheffé test. Moreover, we computed the percentage of poor sleepers with reference to a PSQI-K score of 5 [21]. To identify relationships among the variables, Pearson’s correlation coefficients were calculated, multiple regression analysis was used to identify the factors that affect wellness, and hierarchical multiple regression analysis was used to evaluate the moderating effect of nursing practice environment on the relationship between sleep quality and wellness. Model 1 only included the general characteristics of the participants as predictors of wellness, and Model 2 included sleep quality and nursing practice environment components, along with the general characteristics. Finally, Model 3 included the interaction between sleep quality and nursing practice environment. If the explanatory power of the moderator is significant in Model 3, and the model has a significantly higher explanatory power (R^2^) than that of Model 2, the moderator can be said to have a moderating effect. The adjusted R^2^ of the final regression model was 0.237, indicating a good explanatory power, and the Durbin–Watson coefficient was 2.04, confirming absence of autocorrelation.

## 3. Results

Table 1 shows the participants’ characteristics and the differences in the scores for sleep quality, nursing practice environment, and wellness according to the general characteristics of the participants. Most of the participants were female (95.3%), and the sleep quality of the female participants was poorer than that of male participants (t = 2.10, *p* = 0.036). Regarding education level, 68.3% of the participants had at least a bachelor’s degree. The sleep quality of these participants was higher than that of those with an associate degree (t = −2.39, *p* = 0.017). Almost three-fourths (73.5%) of the participants were either unmarried or divorced, and they reported poorer sleep quality than married participants (t = 3.44, *p* < 0.001). Additionally, 73.9% of the participants worked at medical institutions in the capital region, and there were no significant differences in the sleep quality, nursing practice environment, and wellness between those in the capital region and noncapital region. Regarding the questions on the perceived health status, 61.5% of the participants perceived that they were healthy and reported significantly better sleep quality than those who were not (t = 16.07, *p <* 0.001). Although nurses with less than 1 year of experience had poorer sleep quality than other work experience groups, the difference was not statistically significant (F = 0.83, *p* = 0.480). The ratings of the nursing practice environment differed significantly by gender and years of nursing experience. Female participants had a largely negative evaluation of the nursing practice environment compared to male participants (t = −2.04, *p* = 0.042), and participants with less than 1 year of nursing experience had a more positive view of the nursing practice environment than those in other work experience groups (F = 11.53, *p <* 0.001). Participants who perceived themselves as healthy had a positive perspective of the nursing practice environment (t = 11.00, *p* < 0.001). The wellness level differed significantly by gender, education level, marital status, perceived health status, and nursing experience. The mean wellness score of male participants was higher than that of female participants (t = −4.12, *p* < 0.001). Nurses with at least a bachelor’s degree reported a higher level of wellness than those with an associate degree (t = 3.62, *p* < 0.001). Furthermore, participants who were married and perceived themselves to be healthy had a higher mean wellness score than those who were not married and did not perceive themselves as healthy. With respect to experience, nurses with less than 1 year of experience had a lower wellness score than those with 3–5 years or more than 5 years of experience (F = 3.30, *p* = 0.020).

A total of 90.4% of participants were poor sleepers with a PSQI-K > 5. The mean PSQI-K sleep quality score was 9.37 and was the only factor that demonstrated a negative relationship with nursing practice environment (r = −0.18, *p* < 0.001) and wellness (r = −0.30, *p* < 0.001). Additionally, the mean nursing practice environment score was 2.24, the mean wellness score was 3.07, and nursing practice environment and wellness had a positive relationship (r = 0.28, *p* < 0.001). Upon dividing the nursing practice environment into subcategories, the staffing and resource adequacy score was the lowest, with a mean score of 1.91, followed by participation in facility affairs, with a mean score of 2.10 (Table 2).

**Figure 1:**
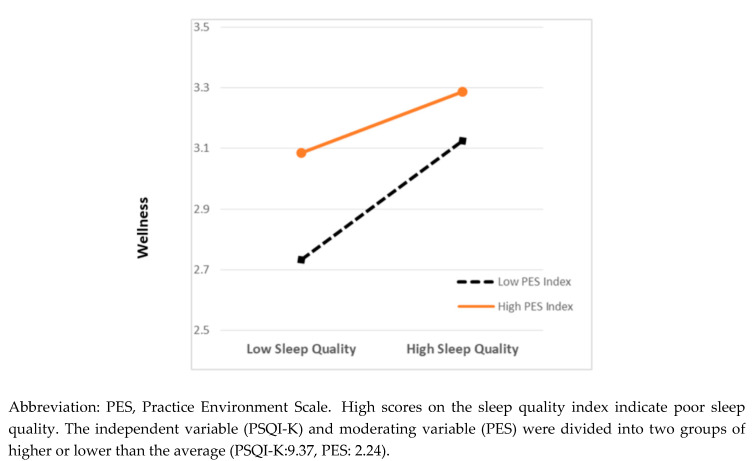
Moderating effect of nursing practice environment in the relationship between nurses’ sleep quality and wellness.

A hierarchical regression analysis was performed to examine the moderating effect of nursing practice environment on the relationship between sleep quality and wellness. The results are shown in Table 3. The results of the multiple regression analysis of Model 1, with only the general characteristics, showed that female participants had lower wellness scores than male participants (*p* = 0.002). It also showed that nurses with at least a bachelor’s degree had higher wellness scores than those with an associate’s degree (*p* < 0.001). Regarding the years of nursing experience, nurses with 3–5 years and more than 5 years of experience had higher wellness scores than nurses with less than 1 year of experience. Furthermore, nurses who perceived themselves as healthy had higher wellness scores than those who did not (*p* < 0.001). In Model 2, the level of wellness decreased with poorer sleep quality (*p* < 0.001), and a more positive evaluation of the nursing practice environment predicted higher wellness scores (*p* < 0.001). The explanatory power was 0.235, which was a substantial increase compared to that of Model 1. When examining the PES-NWI subcategories, of the five factors, participation in facility affairs had a negative association with wellness scores. However, the other four factors showed positive relationships. After adding the interaction of sleep quality and nursing practice environment into the analysis in Model 3, the interaction had a positive relationship with the wellness score (*p =* 0.002), and the change in the adjusted *R*^2^ was also significant (*p* = 0.006). In other words, nursing practice environment was confirmed to have a moderating effect on the relationship between sleep quality and wellness. Thus, these findings indicate that poor sleep quality predicts lower levels of wellness, but the nursing practice environment significantly decreases this negative effect. As the explanatory power of the model increased significantly, a moderating effect was confirmed (Figure 1).

## 4. Discussion

This study aimed to examine the differences in sleep quality, nursing practice environment, and wellness according to the demographic characteristics of nurses. The mean PSQI-K score was 9.37, and 90.4% of the nurses had a score greater than 5. This is descriptively higher than the mean PSQI score among Taiwanese nurses (8.99) [22] and among European nurses (6.8) [23]. Further, in light of the finding that 72.1% of Chinese nurses had a PSQI of greater than 5, Korean nurses seem to have poorer sleep quality compared to their counterparts in other countries [24]. A study on Korean nurses reported that Korean nurses work an average of 43.0 h per week, which is longer than 37.3 h per week among nurses in the US, and working 2.59 weekend days per month [25] seems to worsen their sleep quality. Moreover, we tested the hypothesis that sleep quality is associated with wellness and that the nursing practice environment has a moderating effect on the negative association between poor sleep quality and wellness. Sleep quality and wellness differed according to sex, marital status, and nursing career. Female participants had poorer sleep quality than male participants. This finding is similar to those of previous studies [26,27]. The role of gender in sleep quality may be explained by female participants likely being more susceptible to stress compared to male participants. Female participants may also be expected to undertake more responsibilities at home, which, in turn, may lead to lack of time and have negative emotional effects. Regarding educational level, participants with an associate’s degree had poorer sleep quality than those with at least a bachelor’s degree. This finding is similar to that of a prior study, which showed that lower educational levels are related to higher job stress, burnout, and poorer sleep quality [28,29].

Regarding marital status, unmarried participants had poorer wellness and sleep quality than those who were married, and this finding is also similar to the results of previous studies, which found a positive effect of having a stable married life and a regular family life on sleep quality [30]. Nurses with less than 1 year of nursing experience had poorer sleep quality than those with 3–5 years and more than 5 years of nursing experience. This suggests that nurses with less than 1 year of experience may have poor sleep quality caused by stress from an unfamiliar job and adjusting to a new environment [31].

Regression analysis of the factors that predicted wellness showed that sleep quality is a significant predictor of wellness (*p* < 0.001). Poor sleep quality can have detrimental effects on the harmonization of physical and emotional states, which suggests more efforts should be made to increase the sleep quality of nurses. In particular, interventions need to be developed for female nurses, unmarried nurses, and nurses with limited experience who have a relatively poor quality of sleep. One approach may be to review the shift schedules of newly appointed nurses and continuously monitor nurses’ work intensity.

The mean score for the overall nursing practice environment was 2.24, which is lower than those of general nurses in the United States (3.10) [32] and intensive care unit (ICU) nurses in China (2.99) [33] using the same scale. Considering that a mean score lower than 2.5 indicates a poor nursing practice environment [34], it is evident that nurses in this study had poor nursing practice environments. In particular, the mean score for staffing and resource adequacy was the lowest (1.91), indicating that the nurses in this study felt there was an insufficient number of nurses in hospital wards, which results in lack of time for patient-related discussion and lack of care for patients. Because a sufficient number of nurses is an important factor for patient outcomes, such as patient mortality and adequate nursing care, improving nurse staff is a necessity [35,36].

The multiple regression analysis of the factors predicting wellness showed that better sleep quality and better PES-NWI scores are associated with a higher level of wellness. However, when considering the subcategories of PES-NWI, participation in facility affairs had a negative relationship with wellness. This suggests that lower levels of wellness may be associated with participation in hospital affairs or trying to develop oneself in situations with insufficient staff or resources. A previous study on Japanese nurses reported a low mean PES-NWI score of 1.94 for staffing and resource adequacy, whereas the mean composite score was 2.61. Thus, it should be ensured that there are sufficient staff and resources before providing opportunities to participate in hospital affairs, given that nurse participation in hospital affairs has a negative effect on their ability to provide quality care [37].

The results of the hierarchical regression analysis showed a buffering effect of the nursing practice environment on the negative association between poor sleep quality and perceived wellness. Although there was a negative association between poor sleep quality and overall well-being, it decreased when nurses had a positive impression of their nursing practice environment. Regardless of poor sleep quality due to personal characteristics, negative physical and emotional effects can be decreased with a supportive leadership, sufficient staffing and resources, and a cooperative atmosphere for the members within the organization. This, in turn, can improve the quality of care experienced by patients.

This study examined the buffering effect of the nursing practice environment, which had been largely neglected in the literature in the past. Therefore, subsequent studies should continue to investigate the effect of nursing practice environment on nurses’ retention intentions and job satisfaction. For example, studies should examine the buffering effect of nursing practice environment for factors that have a negative effect on nurses’ retention intentions and job satisfaction. To improve the nursing practice environment, hospitals should implement adequate nursing staffing, and leaders should provide sufficient support for nurses in terms of work and emotional support to ensure that nurses can utilize their resources adequately. Furthermore, governmental policies that obligate health care facilities to implement adequate nursing staffing and provide incentives should be strictly enforced to ensure that these improvements are indeed established.

### Study Limitations and Implications for Health Policy

This study has some limitations. First, the study’s cross-sectional design precludes causal inferences. This study statistically investigated the hypothesis that poor sleep quality is associated with emotional and physical tiredness, which, in turn, can negatively affect the overall wellness of individuals. However, because it is also possible that unstable wellness can negatively affect sleep quality, further longitudinal studies are warranted to determine the exact causal relationship. Second, because most of the measures were subjective and based on the awareness of the participants, future studies should consider measuring the quality of sleep and wellness using objective measures in order to clarify the relationship. Finally, various other factors that could affect wellness, such as the physical health of an individual, were not considered in this study. In particular, we could not collect data on nurses’ shift work and type of shift work despite the fact that they are important predictors of sleep quality. Subsequent studies should examine the differences in sleep quality and wellness according to shift work.

Despite these limitations, this study is significant in shedding light on the importance of nursing practice environment. As nursing practice environment is one of the topics for a national survey in the National Quality Forum and the National Database of Nursing Quality Indicators of the United States, nursing practice environment should be continuously monitored based on which working conditions should be improved.

## 5. Conclusions

The findings of this study indicate that nursing practice environment has a moderating effect on the negative association between nurses’ poor sleep quality and wellness. However, a positive view of the nursing practice environment decreased this association. Furthermore, despite poor sleep quality due to personal characteristics, its negative physical and emotional effects may be reduced with the help of a supportive leadership, adequate staffing and resources, and a cooperative atmosphere for the members within the organization, which, in turn, can improve the quality of nursing services. This study confirmed that nursing practice environment may impact nurses’ quality of life, and that the influence of factors that negatively impact nurses’ health and quality of life can be buffered by ensuring adequate nursing staffing and providing a supportive climate for nurses. Therefore, policies to improve nurses’ well-being should be implemented not only at the individual level but also at the organizational level and nursing work environment. Furthermore, subsequent studies are needed on the role of nursing practice environment in its effect on nurses’ turnover intentions and job satisfaction.

## Figures and Tables

**Table 1:** Sleep quality, nursing practice environment, and wellness by participants’ characteristics (N = 1874).

	*n* (%)	PSQI-K		PES-NWI		WI	
		M ± *SD*	*t or F (p)*	M ± *SD*	*P*	M ± *SD*	*p*
Gender						
Female	1785 (95.3)	9.4 ± 3.3	2.10 (0.036)	2.2 ± 0.5	−2.04 (0.042)	3.1 ± 0.6	−4.12 (<0.001)
Male	89 (4.8)	8.7 ± 2.8		2.3 ± 0.5		3.3 ± 0.7	
Education							
Bachelor’s or higher	1280 (68.3)	9.2 ± 3.2	−2.39 (0.017)	2.2 ± 0.5	1.01 (0.314)	3.1 ± 0.6	3.62 (<0.001)
Associate degree	594 (31.7)	9.6 ± 3.4		2.2 ± 0.5		3.0 ± 0.6	
Marital status							
Single or divorced	1378 (73.5)	9.5 ± 3.3	3.44 (<0.001)	2.2 ± 0.5	0.01 (0.995)	3.0 ± 0.6	−2.48 (0.013)
Married	496 (26.5)	8.9 ± 3.2		2.2 ± 0.5		3.1 ± 0.6	
Region of working institution							
Capital region	1384 (73.9)	9.4 ± 3.3	1.54 (0.124)	2.2 ± 0.5	−0.74 (0.462)	3.1 ± 0.7	−0.10 (0.924)
Noncapital region	490 (26.2)	9.2 ± 3.3		2.3 ± 0.5		3.1 ± 0.6	
Perceived health status							
Healthy	1152 (61.5)	8.5 ± 2.9	16.07 (<0.001)	2.3 ±3.3	11.00 (<0.001)	3.3 ± 3.2	19.66 (<0.001)
Unhealthy	722 (38.5)	10.8 ± 3.3		2.1 ± 2.9		2.7 ± 2.7	
Years worked as a registered nurse						
(1) < 1 ^a^	133 (7.1)	9.8 ± 3.0	0.83 (0.480)	2.5 ± 0.5	11.53 (<0.001) a < b,c,d	2.9 ± 0.7	3.30 (0.020) a < c,d
(2) 1− < 3 ^b^	402 (21.5)	9.4 ± 3.4		2.3 ± 0.5		3.1 ± 0.6	
(3) 3− < 5 ^c^	544 (29.0)	9.4 ± 3.4		2.2 ± 0.5		3.1 ± 0.6	
(4) ≥ 5 ^d^	795 (42.4)	9.3 ± 3.2		2.2 ± 0.5		3.1 ± 0.6	

High scores on the sleep quality index indicate poor sleep quality.

**Table 2:** Descriptive statistics and Pearson’s correlations among variables (N = 1874).

Variables	M ± *SD/n(%)*	Range (min, max)	Pearson Correlation	
			PSQI-K	WI
Poor sleepers (PSQI-K > 5)	1694 (90.4)			
PSQI-K (M ± SD)	9.37 ± 3.31	0–21 (2, 21)		−0.30 (<0.001)
PES-NWI (total composite score)	2.24 ± 0.49	1–4 (1, 4)	−0.18 (<0.001)	0.28 (<0.001)
Participation in facility affairs	2.10 ± 0.56	1–4 (1, 4)	−0.16 (<0.001)	0.21 (<0.001)
Foundation for quality of care	2.43 ± 0.53	1–4 (1, 4)	−0.14 (<0.001)	0.28 (<0.001)
Resource adequacy	1.91 ± 0.59	1–4 (1, 4)	−0.17 (<0.001)	0.22 (<0.001)
Supportive manager	2.34 ± 0.68	1–4 (1, 4)	−0.16 (<0.001)	0.22 (<0.001)
Nurse–physician relationship	2.40 ± 0.64	1–4 (1, 4)	−0.12 (<0.001)	0.22 (<0.001)
WI	3.07 ± 0.64	1–5 (1, 5)	−0.30 (<0.001)	

High scores on the sleep quality index indicate poor sleep quality.

**Table 3:** Results of the hierarchical multiple regression analyses predicting wellness (N = 1874).

Dependent Variable		WI		
	Model 1	Model 2 (Composite)	Model 2 (Subscales)	Model 3
	β (*p)*	β (*p)*	β (*p)*	β (*p)*
Female (vs. male)	−0.06 (0.002)	−0.06 (0.005)	−0.06 (0.005)	−0.58 (0.004)
Bachelor’s or higher (vs. associate’s)	0.09 (<0.001)	0.08 (<0.001)	0.07 (<0.001)	0.08 (<0.001)
Married (vs. not)	−0.04 (0.074)	−0.03 (0.227)	−0.02 (0.244)	−0.03 (0.235)
Region of working institution (vs. capital region)	0.00(0.973)	−0.01(0.656)	0.00(0.842)	−0.01 (0.632)
Experience as RN (vs. < 1 year)				
1 to < 3	0.06 (0.087)	0.10 (0.006)	0.10 (0.005)	0.10 (0.005)
3 to < 5	0.10 (0.016)	0.15 (<0.001)	0.14 (<0.001)	0.15 (<0.001)
≥5	0.10 (0.027)	0.15 (0.001)	0.14 (0.001)	0.15 (<0.001)
Perceived health status (vs. unhealthy)	0.40 (<0.001)	0.31 (<0.001)	0.30 (<0.001)	0.30 (<0.001)
PSQI-K		−0.15 (<0.001)	−0.13 (<0.001)	−0.34 (<0.001)
PES-NWI (composite)		0.18 (<0.001)		0.06(0.318)
Participation in facility affairs			−0.15 (<0.001)	
Foundation for the quality of care			0.21 (<0.001)	
Resource adequacy			0.07 (0.014)	
Supportive manager			0.05 (0.073)	
Nurse-physician relationship			0.07 (0.008)	
PSQI-K × PES-NWI				0.21 (0.028)
*F*	53.18	58.64	45.48	53.85
Adjusted *R*^2^	0.182	0.235	0.250	0.237
Δ Adjusted *R*^2^ (*p*)		0.053	0.068	0.002 (0.006)

Model 1: Only included the general characteristics of the participants as predictors of wellness. Model 2: Sleep quality and nursing practice environment components were added. Model 3: The interaction term between sleep quality and nursing practice environment was added.

## Abbreviations

PES: Practice Environment Scale. * High scores on the sleep quality index indicate poor sleep quality. * The independent variable (PSQI-K) and moderating variable (PES) were divided into two groups of higher or lower than the average (PSQI-K:9.37, PES: 2.24).
